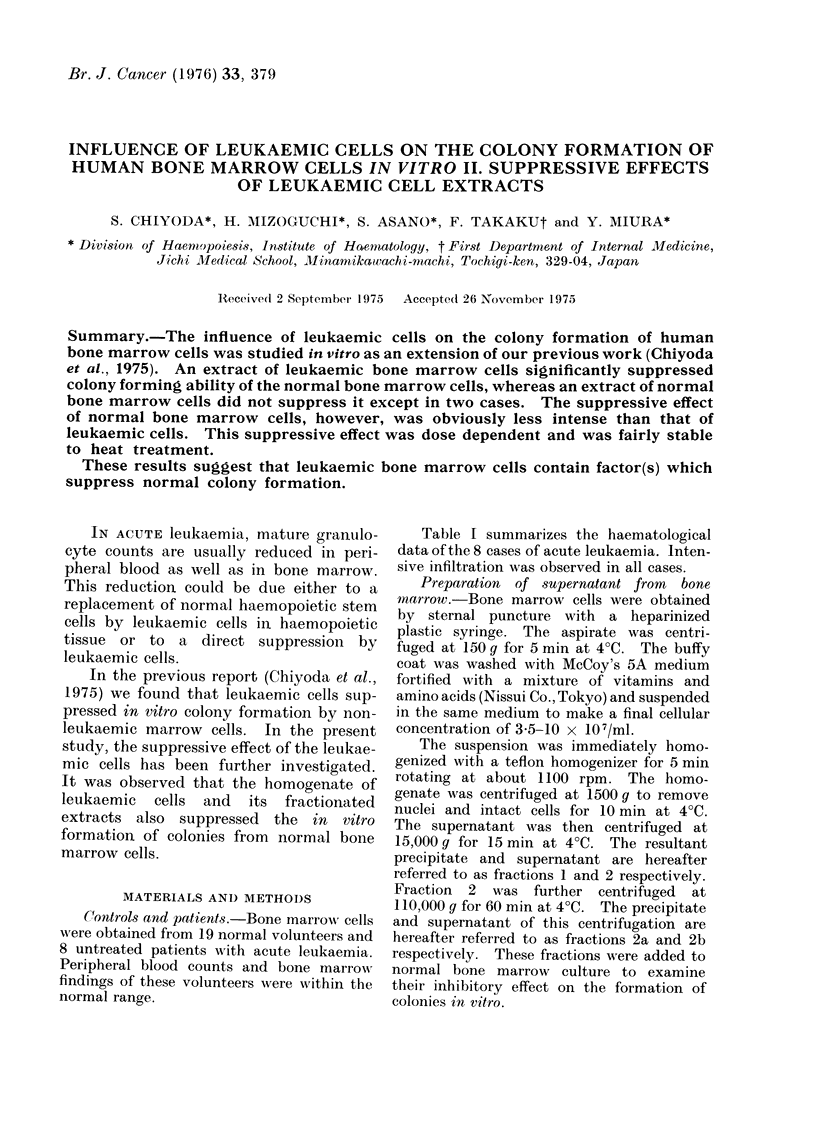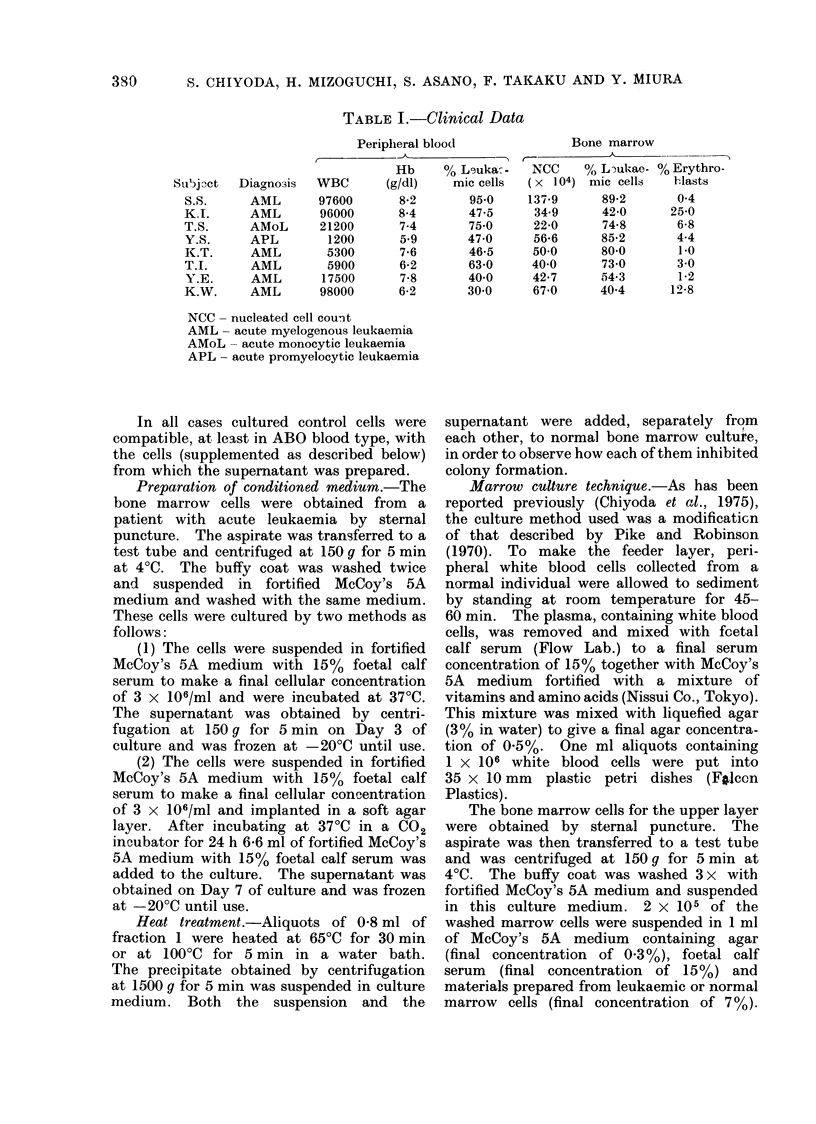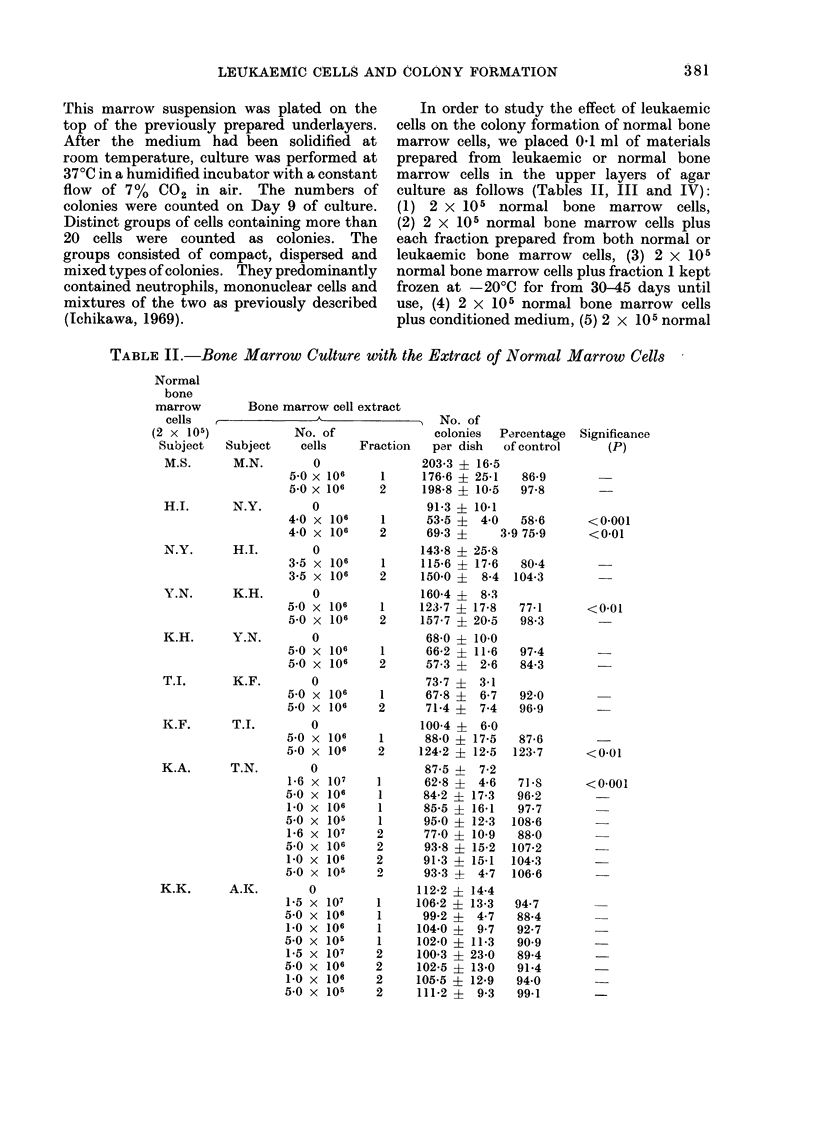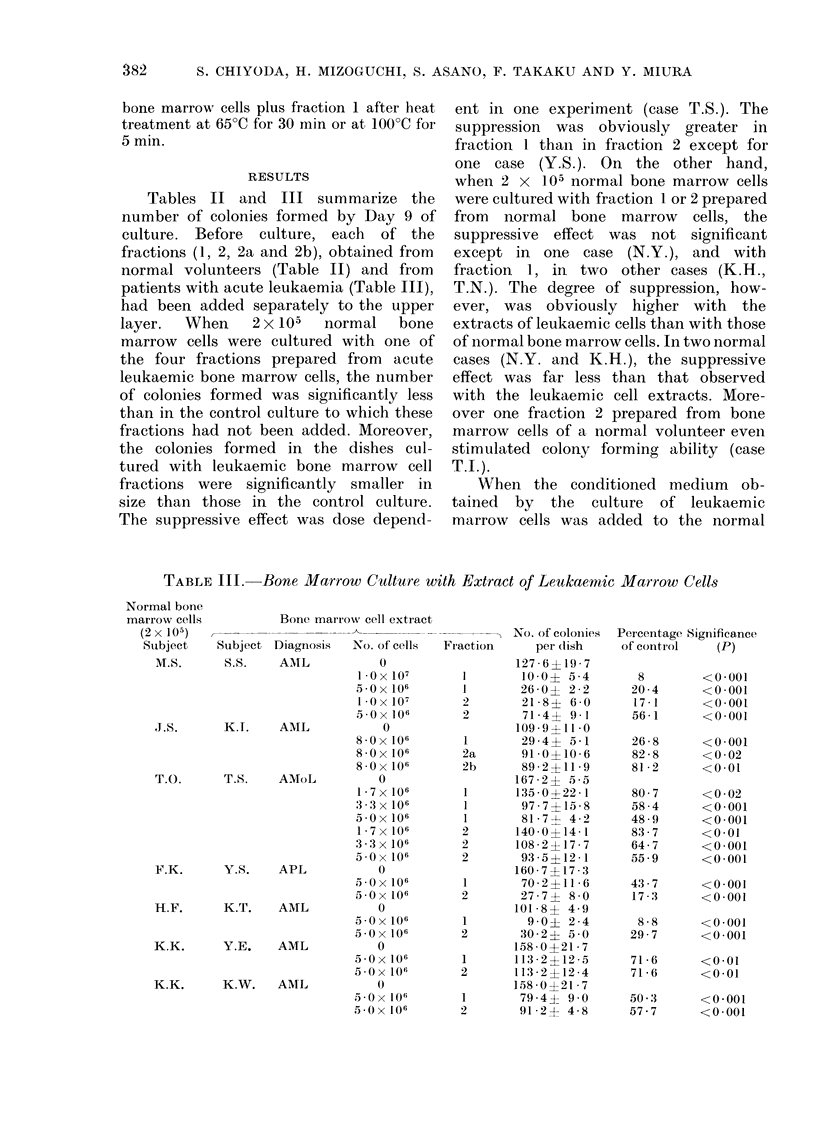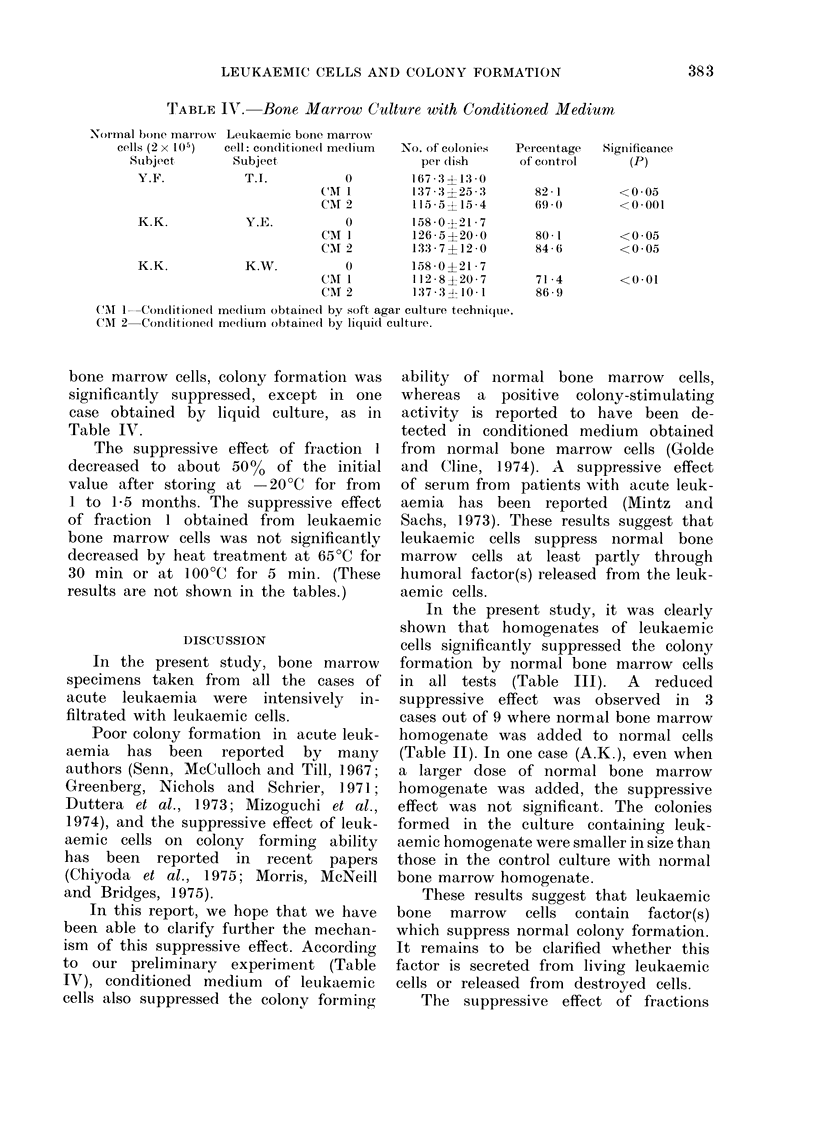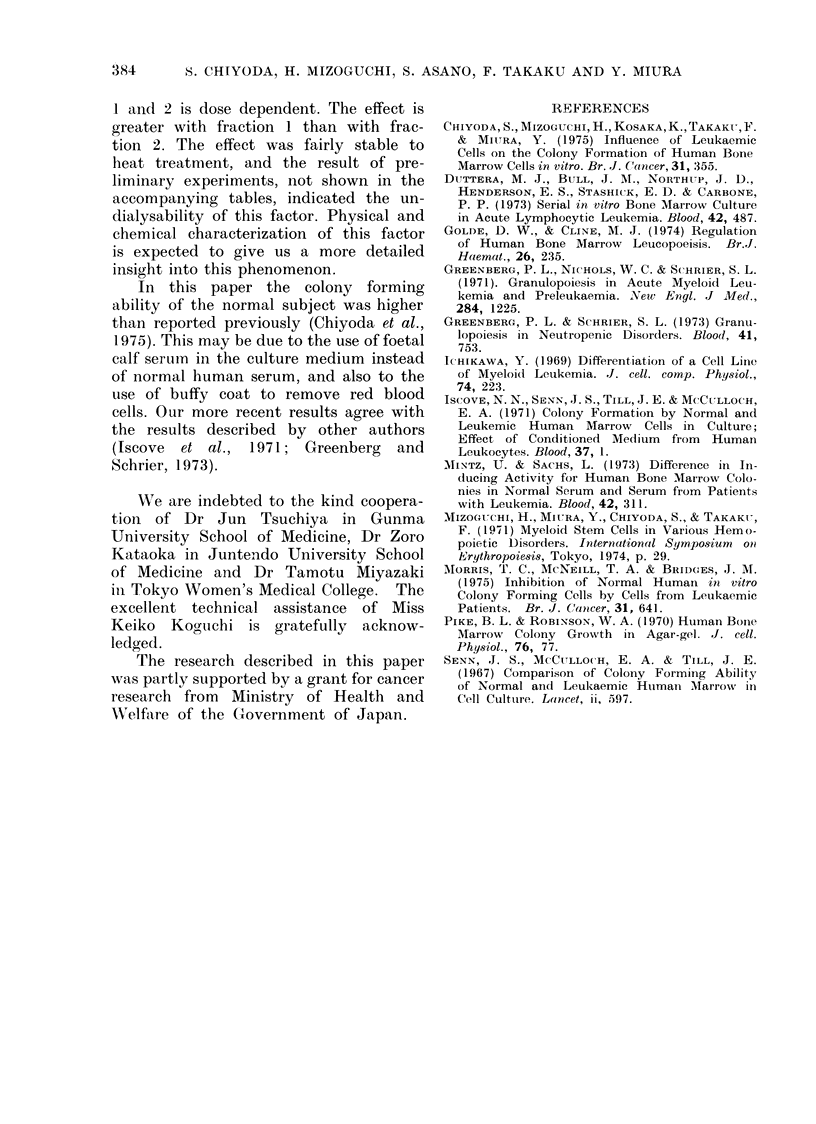# Influence of leukaemic cells on the colony formation of human bone marrow cells in vitro II. Suppressive effects of leukaemic cell extracts.

**DOI:** 10.1038/bjc.1976.62

**Published:** 1976-04

**Authors:** S. Chiyoda, H. Mizoguchi, S. Asano, F. Takaku, Y. Miura

## Abstract

The influence of leukaemic cells on the colony formation of human bone marrow cells was studied in vitro as an extension of our previous work (Chiyoda et al., 1975). An extract of leukaemic bone marrow cells significantly suppressed colony forming ability of the normal bone marrow cells, whereas an extract of normal bone marrow cells did not suppress it except in two cases. The suppressive effect of normal bone marrow cells, however, was obviously less intense than that of leukaemic cells. This suppressive effect was dose dependent and was fairly stable to heat treatment. These results suggest that leukaemic bone marrow cells contain factor(s) which suppress normal colony formation.


*Br. J. Cancer* (1976) **33**, 379

### INFLUENCE OF LEUKAEMIC CELLS ON THE COLONY FORMATION OF HUMAN BONE MARROW CELLS *IN VITRO* II. SUPPRESSIVE EFFECTS OF LEUKAEMIC CELL EXTRACTS

S. CHIYODA*, H. MIZOGUCHI*, S. ASANO*, F. TAKAKU† and Y. MIURA*

* *Division of Haemopoiesis, Institute of Haematology,* † *First Department of Internal Medicine, Jichi Medical School, Minamikawachi-machi, Tochigi-ken,* 329-04, *Japan*

Received 2 September 1975 Accepted 26 November 1975

**Summary.—The influence of leukaemic cells on the colony formation of human bone marrow cells was studied *in vitro* as an extension of our previous work (Chiyoda *et al.*, 1975). An extract of leukaemic bone marrow cells significantly suppressed colony forming ability of the normal bone marrow cells, whereas an extract of normal bone marrow cells did not suppress it except in two cases. The suppressive effect of normal bone marrow cells, however, was obviously less intense than that of leukaemic cells. This suppressive effect was dose dependent and was fairly stable to heat treatment.**

**These results suggest that leukaemic bone marrow cells contain factor(s) which suppress normal colony formation.**

In ACUTE leukaemia, mature granulocyte counts are usually reduced in peripheral blood as well as in bone marrow. This reduction could be due either to a replacement of normal haemopoietic stem cells by leukaemic cells in haemopoietic tissue or to a direct suppression by leukaemic cells.

In the previous report (Chiyoda *et al.*, 1975) we found that leukaemic cells suppressed *in vitro* colony formation by non-leukaemic marrow cells. In the present study, the suppressive effect of the leukaemic cells has been further investigated. It was observed that the homogenate of leukaemic cells and its fractionated extracts also suppressed the *in vitro* formation of colonies from normal bone marrow cells.

### MATERIALS AND METHODS

*Controls and patients.*—Bone marrow cells were obtained from 19 normal volunteers and 8 untreated patients with acute leukaemia. Peripheral blood counts and bone marrow findings of these volunteers were within the normal range.

Table I summarizes the haematological data of the 8 cases of acute leukaemia. Intensive infiltration was observed in all cases.

*Preparation of supernatant from bone marrow.*—Bone marrow cells were obtained by sternal puncture with a heparinized plastic syringe. The aspirate was centrifuged at 150 *g* for 5 min at 4°C. The buffy coat was washed with McCoy's 5A medium fortified with a mixture of vitamins and amino acids (Nissui Co., Tokyo) and suspended in the same medium to make a final cellular concentration of 3·5–10 × $10^7$/ml.

The suspension was immediately homogenized with a teflon homogenizer for 5 min rotating at about 1100 rpm. The homogenate was centrifuged at 1500 *g* to remove nuclei and intact cells for 10 min at 4°C. The supernatant was then centrifuged at 15,000 *g* for 15 min at 4°C. The resultant precipitate and supernatant are hereafter referred to as fractions 1 and 2 respectively. Fraction 2 was further centrifuged at 110,000 *g* for 60 min at 4°C. The precipitate and supernatant of this centrifugation are hereafter referred to as fractions 2a and 2b respectively. These fractions were added to normal bone marrow culture to examine their inhibitory effect on the formation of colonies *in vitro*.

TABLE I.—*Clinical Data*

| | | Peripheral blood | | | Bone marrow | | |
|---|---|---|---|---|---|---|---|
| Subject | Diagnosis | WBC | Hb (g/dl) | % Leukaemic cells | NCC ($\times 10^4$) | % Leukaemic cells | % Erythroblasts |
| S.S. | AML | 97600 | 8·2 | 95·0 | 137·9 | 89·2 | 0·4 |
| K.I. | AML | 96000 | 8·4 | 47·5 | 34·9 | 42·0 | 25·0 |
| T.S. | AMoL | 21200 | 7·4 | 75·0 | 22·0 | 74·8 | 6·8 |
| Y.S. | APL | 1200 | 5·9 | 47·0 | 56·6 | 85·2 | 4·4 |
| K.T. | AML | 5300 | 7·6 | 46·5 | 50·0 | 80·0 | 1·0 |
| T.I. | AML | 5900 | 6·2 | 63·0 | 40·0 | 73·0 | 3·0 |
| Y.E. | AML | 17500 | 7·8 | 40·0 | 42·7 | 54·3 | 1·2 |
| K.W. | AML | 98000 | 6·2 | 30·0 | 67·0 | 40·4 | 12·8 |

NCC – nucleated cell count
AML – acute myelogenous leukaemia
AMoL – acute monocytic leukaemia
APL – acute promyelocytic leukaemia

In all cases cultured control cells were compatible, at least in ABO blood type, with the cells (supplemented as described below) from which the supernatant was prepared.

*Preparation of conditioned medium.*—The bone marrow cells were obtained from a patient with acute leukaemia by sternal puncture. The aspirate was transferred to a test tube and centrifuged at 150 *g* for 5 min at 4°C. The buffy coat was washed twice and suspended in fortified McCoy's 5A medium and washed with the same medium. These cells were cultured by two methods as follows:

(1) The cells were suspended in fortified McCoy's 5A medium with 15% foetal calf serum to make a final cellular concentration of $3 \times 10^6$/ml and were incubated at 37°C. The supernatant was obtained by centrifugation at 150 *g* for 5 min on Day 3 of culture and was frozen at −20°C until use.

(2) The cells were suspended in fortified McCoy's 5A medium with 15% foetal calf serum to make a final cellular concentration of $3 \times 10^6$/ml and implanted in a soft agar layer. After incubating at 37°C in a $CO_2$ incubator for 24 h 6·6 ml of fortified McCoy's 5A medium with 15% foetal calf serum was added to the culture. The supernatant was obtained on Day 7 of culture and was frozen at −20°C until use.

*Heat treatment.*—Aliquots of 0·8 ml of fraction 1 were heated at 65°C for 30 min or at 100°C for 5 min in a water bath. The precipitate obtained by centrifugation at 1500 *g* for 5 min was suspended in culture medium. Both the suspension and the supernatant were added, separately from each other, to normal bone marrow culture, in order to observe how each of them inhibited colony formation.

*Marrow culture technique.*—As has been reported previously (Chiyoda *et al.*, 1975), the culture method used was a modification of that described by Pike and Robinson (1970). To make the feeder layer, peripheral white blood cells collected from a normal individual were allowed to sediment by standing at room temperature for 45–60 min. The plasma, containing white blood cells, was removed and mixed with foetal calf serum (Flow Lab.) to a final serum concentration of 15% together with McCoy's 5A medium fortified with a mixture of vitamins and amino acids (Nissui Co., Tokyo). This mixture was mixed with liquefied agar (3% in water) to give a final agar concentration of 0·5%. One ml aliquots containing $1 \times 10^6$ white blood cells were put into 35 × 10 mm plastic petri dishes (Falcon Plastics).

The bone marrow cells for the upper layer were obtained by sternal puncture. The aspirate was then transferred to a test tube and was centrifuged at 150 *g* for 5 min at 4°C. The buffy coat was washed 3× with fortified McCoy's 5A medium and suspended in this culture medium. $2 \times 10^5$ of the washed marrow cells were suspended in 1 ml of McCoy's 5A medium containing agar (final concentration of 0·3%), foetal calf serum (final concentration of 15%) and materials prepared from leukaemic or normal marrow cells (final concentration of 7%).

This marrow suspension was plated on the top of the previously prepared underlayers. After the medium had been solidified at room temperature, culture was performed at 37°C in a humidified incubator with a constant flow of 7% $CO_2$ in air. The numbers of colonies were counted on Day 9 of culture. Distinct groups of cells containing more than 20 cells were counted as colonies. The groups consisted of compact, dispersed and mixed types of colonies. They predominantly contained neutrophils, mononuclear cells and mixtures of the two as previously described (Ichikawa, 1969).

In order to study the effect of leukaemic cells on the colony formation of normal bone marrow cells, we placed 0·1 ml of materials prepared from leukaemic or normal bone marrow cells in the upper layers of agar culture as follows (Tables II, III and IV): (1) $2 \times 10^5$ normal bone marrow cells, (2) $2 \times 10^5$ normal bone marrow cells plus each fraction prepared from both normal or leukaemic bone marrow cells, (3) $2 \times 10^5$ normal bone marrow cells plus fraction 1 kept frozen at −20°C for from 30–45 days until use, (4) $2 \times 10^5$ normal bone marrow cells plus conditioned medium, (5) $2 \times 10^5$ normal

TABLE II.—*Bone Marrow Culture with the Extract of Normal Marrow Cells*

| Normal bone marrow cells ($2 \times 10^5$) Subject | Bone marrow cell extract: Subject | No. of cells | Fraction | No. of colonies per dish | Percentage of control | Significance ($P$) |
|---|---|---|---|---|---|---|
| M.S. | M.N. | 0 | | 203·3 ± 16·5 | | |
| | | $5·0 \times 10^6$ | 1 | 176·6 ± 25·1 | 86·9 | — |
| | | $5·0 \times 10^6$ | 2 | 198·8 ± 10·5 | 97·8 | — |
| H.I. | N.Y. | 0 | | 91·3 ± 10·1 | | |
| | | $4·0 \times 10^6$ | 1 | 53·5 ± 4·0 | 58·6 | <0·001 |
| | | $4·0 \times 10^6$ | 2 | 69·3 ± 3·9 | 75·9 | <0·01 |
| N.Y. | H.I. | 0 | | 143·8 ± 25·8 | | |
| | | $3·5 \times 10^6$ | 1 | 115·6 ± 17·6 | 80·4 | — |
| | | $3·5 \times 10^6$ | 2 | 150·0 ± 8·4 | 104·3 | — |
| Y.N. | K.H. | 0 | | 160·4 ± 8·3 | | |
| | | $5·0 \times 10^6$ | 1 | 123·7 ± 17·8 | 77·1 | <0·01 |
| | | $5·0 \times 10^6$ | 2 | 157·7 ± 20·5 | 98·3 | — |
| K.H. | Y.N. | 0 | | 68·0 ± 10·0 | | |
| | | $5·0 \times 10^6$ | 1 | 66·2 ± 11·6 | 97·4 | — |
| | | $5·0 \times 10^6$ | 2 | 57·3 ± 2·6 | 84·3 | — |
| T.I. | K.F. | 0 | | 73·7 ± 3·1 | | |
| | | $5·0 \times 10^6$ | 1 | 67·8 ± 6·7 | 92·0 | — |
| | | $5·0 \times 10^6$ | 2 | 71·4 ± 7·4 | 96·9 | — |
| K.F. | T.I. | 0 | | 100·4 ± 6·0 | | |
| | | $5·0 \times 10^6$ | 1 | 88·0 ± 17·5 | 87·6 | — |
| | | $5·0 \times 10^6$ | 2 | 124·2 ± 12·5 | 123·7 | <0·01 |
| K.A. | T.N. | 0 | | 87·5 ± 7·2 | | |
| | | $1·6 \times 10^7$ | 1 | 62·8 ± 4·6 | 71·8 | <0·001 |
| | | $5·0 \times 10^6$ | 1 | 84·2 ± 17·3 | 96·2 | — |
| | | $1·0 \times 10^6$ | 1 | 85·5 ± 16·1 | 97·7 | — |
| | | $5·0 \times 10^5$ | 1 | 95·0 ± 12·3 | 108·6 | — |
| | | $1·6 \times 10^7$ | 2 | 77·0 ± 10·9 | 88·0 | — |
| | | $5·0 \times 10^6$ | 2 | 93·8 ± 15·2 | 107·2 | — |
| | | $1·0 \times 10^6$ | 2 | 91·3 ± 15·1 | 104·3 | — |
| | | $5·0 \times 10^5$ | 2 | 93·3 ± 4·7 | 106·6 | — |
| K.K. | A.K. | 0 | | 112·2 ± 14·4 | | |
| | | $1·5 \times 10^7$ | 1 | 106·2 ± 13·3 | 94·7 | — |
| | | $5·0 \times 10^6$ | 1 | 99·2 ± 4·7 | 88·4 | — |
| | | $1·0 \times 10^6$ | 1 | 104·0 ± 9·7 | 92·7 | — |
| | | $5·0 \times 10^5$ | 1 | 102·0 ± 11·3 | 90·9 | — |
| | | $1·5 \times 10^7$ | 2 | 100·3 ± 23·0 | 89·4 | — |
| | | $5·0 \times 10^6$ | 2 | 102·5 ± 13·0 | 91·4 | — |
| | | $1·0 \times 10^6$ | 2 | 105·5 ± 12·9 | 94·0 | — |
| | | $5·0 \times 10^5$ | 2 | 111·2 ± 9·3 | 99·1 | — |

bone marrow cells plus fraction 1 after heat treatment at 65°C for 30 min or at 100°C for 5 min.

### RESULTS

Tables II and III summarize the number of colonies formed by Day 9 of culture. Before culture, each of the fractions (1, 2, 2a and 2b), obtained from normal volunteers (Table II) and from patients with acute leukaemia (Table III), had been added separately to the upper layer. When $2 \times 10^5$ normal bone marrow cells were cultured with one of the four fractions prepared from acute leukaemic bone marrow cells, the number of colonies formed was significantly less than in the control culture to which these fractions had not been added. Moreover, the colonies formed in the dishes cultured with leukaemic bone marrow cell fractions were significantly smaller in size than those in the control culture. The suppressive effect was dose dependent in one experiment (case T.S.). The suppression was obviously greater in fraction 1 than in fraction 2 except for one case (Y.S.). On the other hand, when $2 \times 10^5$ normal bone marrow cells were cultured with fraction 1 or 2 prepared from normal bone marrow cells, the suppressive effect was not significant except in one case (N.Y.), and with fraction 1, in two other cases (K.H., T.N.). The degree of suppression, however, was obviously higher with the extracts of leukaemic cells than with those of normal bone marrow cells. In two normal cases (N.Y. and K.H.), the suppressive effect was far less than that observed with the leukaemic cell extracts. Moreover one fraction 2 prepared from bone marrow cells of a normal volunteer even stimulated colony forming ability (case T.I.).

When the conditioned medium obtained by the culture of leukaemic marrow cells was added to the normal

TABLE III.—*Bone Marrow Culture with Extract of Leukaemic Marrow Cells*

| Normal bone marrow cells ($2 \times 10^5$) Subject | Bone marrow cell extract Subject | Diagnosis | No. of cells | Fraction | No. of colonies per dish | Percentage of control | Significance (*P*) |
|---|---|---|---|---|---|---|---|
| M.S. | S.S. | AML | 0 | | 127·6±19·7 | | |
| | | | $1 \cdot 0 \times 10^7$ | 1 | 10·0± 5·4 | 8 | <0·001 |
| | | | $5 \cdot 0 \times 10^6$ | 1 | 26·0± 2·2 | 20·4 | <0·001 |
| | | | $1 \cdot 0 \times 10^7$ | 2 | 21·8± 6·0 | 17·1 | <0·001 |
| | | | $5 \cdot 0 \times 10^6$ | 2 | 71·4± 9·1 | 56·1 | <0·001 |
| J.S. | K.I. | AML | 0 | | 109·9±11·0 | | |
| | | | $8 \cdot 0 \times 10^6$ | 1 | 29·4± 5·1 | 26·8 | <0·001 |
| | | | $8 \cdot 0 \times 10^6$ | 2a | 91·0±10·6 | 82·8 | <0·02 |
| | | | $8 \cdot 0 \times 10^6$ | 2b | 89·2±11·9 | 81·2 | <0·01 |
| T.O. | T.S. | AMoL | 0 | | 167·2± 5·5 | | |
| | | | $1 \cdot 7 \times 10^6$ | 1 | 135·0±22·1 | 80·7 | <0·02 |
| | | | $3 \cdot 3 \times 10^6$ | 1 | 97·7±15·8 | 58·4 | <0·001 |
| | | | $5 \cdot 0 \times 10^6$ | 1 | 81·7± 4·2 | 48·9 | <0·001 |
| | | | $1 \cdot 7 \times 10^6$ | 2 | 140·0±14·1 | 83·7 | <0·01 |
| | | | $3 \cdot 3 \times 10^6$ | 2 | 108·2±17·7 | 64·7 | <0·001 |
| | | | $5 \cdot 0 \times 10^6$ | 2 | 93·5±12·1 | 55·9 | <0·001 |
| F.K. | Y.S. | APL | 0 | | 160·7±17·3 | | |
| | | | $5 \cdot 0 \times 10^6$ | 1 | 70·2±11·6 | 43·7 | <0·001 |
| | | | $5 \cdot 0 \times 10^6$ | 2 | 27·7± 8·0 | 17·3 | <0·001 |
| H.F. | K.T. | AML | 0 | | 101·8± 4·9 | | |
| | | | $5 \cdot 0 \times 10^6$ | 1 | 9·0± 2·4 | 8·8 | <0·001 |
| | | | $5 \cdot 0 \times 10^6$ | 2 | 30·2± 5·0 | 29·7 | <0·001 |
| K.K. | Y.E. | AML | 0 | | 158·0±21·7 | | |
| | | | $5 \cdot 0 \times 10^6$ | 1 | 113·2±12·5 | 71·6 | <0·01 |
| | | | $5 \cdot 0 \times 10^6$ | 2 | 113·2±12·4 | 71·6 | <0·01 |
| K.K. | K.W. | AML | 0 | | 158·0±21·7 | | |
| | | | $5 \cdot 0 \times 10^6$ | 1 | 79·4± 9·0 | 50·3 | <0·001 |
| | | | $5 \cdot 0 \times 10^6$ | 2 | 91·2± 4·8 | 57·7 | <0·001 |

TABLE IV.—*Bone Marrow Culture with Conditioned Medium*

| Normal bone marrow cells ($2 \times 10^5$) Subject | Leukaemic bone marrow cell: conditioned medium Subject | | No. of colonies per dish | Percentage of control | Significance ($P$) |
|---|---|---|---|---|---|
| Y.F. | T.I. | 0 | 167·3±13·0 | | |
| | | CM 1 | 137·3±25·3 | 82·1 | <0·05 |
| | | CM 2 | 115·5±15·4 | 69·0 | <0·001 |
| K.K. | Y.E. | 0 | 158·0±21·7 | | |
| | | CM 1 | 126·5±20·0 | 80·1 | <0·05 |
| | | CM 2 | 133·7±12·0 | 84·6 | <0·05 |
| K.K. | K.W. | 0 | 158·0±21·7 | | |
| | | CM 1 | 112·8±20·7 | 71·4 | <0·01 |
| | | CM 2 | 137·3±10·1 | 86·9 | |

CM 1—Conditioned medium obtained by soft agar culture technique.
CM 2—Conditioned medium obtained by liquid culture.

bone marrow cells, colony formation was significantly suppressed, except in one case obtained by liquid culture, as in Table IV.

The suppressive effect of fraction 1 decreased to about 50% of the initial value after storing at −20°C for from 1 to 1·5 months. The suppressive effect of fraction 1 obtained from leukaemic bone marrow cells was not significantly decreased by heat treatment at 65°C for 30 min or at 100°C for 5 min. (These results are not shown in the tables.)

### DISCUSSION

In the present study, bone marrow specimens taken from all the cases of acute leukaemia were intensively infiltrated with leukaemic cells.

Poor colony formation in acute leukaemia has been reported by many authors (Senn, McCulloch and Till, 1967; Greenberg, Nichols and Schrier, 1971; Duttera *et al.*, 1973; Mizoguchi *et al.*, 1974), and the suppressive effect of leukaemic cells on colony forming ability has been reported in recent papers (Chiyoda *et al.*, 1975; Morris, McNeill and Bridges, 1975).

In this report, we hope that we have been able to clarify further the mechanism of this suppressive effect. According to our preliminary experiment (Table IV), conditioned medium of leukaemic cells also suppressed the colony forming ability of normal bone marrow cells, whereas a positive colony-stimulating activity is reported to have been detected in conditioned medium obtained from normal bone marrow cells (Golde and Cline, 1974). A suppressive effect of serum from patients with acute leukaemia has been reported (Mintz and Sachs, 1973). These results suggest that leukaemic cells suppress normal bone marrow cells at least partly through humoral factor(s) released from the leukaemic cells.

In the present study, it was clearly shown that homogenates of leukaemic cells significantly suppressed the colony formation by normal bone marrow cells in all tests (Table III). A reduced suppressive effect was observed in 3 cases out of 9 where normal bone marrow homogenate was added to normal cells (Table II). In one case (A.K.), even when a larger dose of normal bone marrow homogenate was added, the suppressive effect was not significant. The colonies formed in the culture containing leukaemic homogenate were smaller in size than those in the control culture with normal bone marrow homogenate.

These results suggest that leukaemic bone marrow cells contain factor(s) which suppress normal colony formation. It remains to be clarified whether this factor is secreted from living leukaemic cells or released from destroyed cells.

The suppressive effect of fractions

1 and 2 is dose dependent. The effect is greater with fraction 1 than with fraction 2. The effect was fairly stable to heat treatment, and the result of preliminary experiments, not shown in the accompanying tables, indicated the undialysability of this factor. Physical and chemical characterization of this factor is expected to give us a more detailed insight into this phenomenon.

In this paper the colony forming ability of the normal subject was higher than reported previously (Chiyoda *et al.*, 1975). This may be due to the use of foetal calf serum in the culture medium instead of normal human serum, and also to the use of buffy coat to remove red blood cells. Our more recent results agree with the results described by other authors (Iscove *et al.*, 1971; Greenberg and Schrier, 1973).

We are indebted to the kind cooperation of Dr Jun Tsuchiya in Gunma University School of Medicine, Dr Zoro Kataoka in Juntendo University School of Medicine and Dr Tamotu Miyazaki in Tokyo Women's Medical College. The excellent technical assistance of Miss Keiko Koguchi is gratefully acknowledged.

The research described in this paper was partly supported by a grant for cancer research from Ministry of Health and Welfare of the Government of Japan.

REFERENCES

CHIYODA, S., MIZOGUCHI, H., KOSAKA, K., TAKAKU, F. & MIURA, Y. (1975) Influence of Leukaemic Cells on the Colony Formation of Human Bone Marrow Cells *in vitro*. *Br. J. Cancer*, **31**, 355.

DUTTERA, M. J., BULL, J. M., NORTHUP, J. D., HENDERSON, E. S., STASHICK, E. D. & CARBONE, P. P. (1973) Serial *in vitro* Bone Marrow Culture in Acute Lymphocytic Leukemia. *Blood*, **42**, 487.

GOLDE, D. W., & CLINE, M. J. (1974) Regulation of Human Bone Marrow Leucopoeisis. *Br.J. Haemat.*, **26**, 235.

GREENBERG, P. L., NICHOLS, W. C. & SCHRIER, S. L. (1971). Granulopoiesis in Acute Myeloid Leukemia and Preleukaemia. *New Engl. J Med.*, **284**, 1225.

GREENBERG, P. L. & SCHRIER, S. L. (1973) Granulopoiesis in Neutropenic Disorders. *Blood*, **41**, 753.

ICHIKAWA, Y. (1969) Differentiation of a Cell Line of Myeloid Leukemia. *J. cell. comp. Physiol.*, **74**, 223.

ISCOVE, N. N., SENN, J. S., TILL, J. E. & McCULLOCH, E. A. (1971) Colony Formation by Normal and Leukemic Human Marrow Cells in Culture; Effect of Conditioned Medium from Human Leukocytes. *Blood*, **37**, 1.

MINTZ, U. & SACHS, L. (1973) Difference in Inducing Activity for Human Bone Marrow Colonies in Normal Serum and Serum from Patients with Leukemia. *Blood*, **42**, 311.

MIZOGUCHI, H., MIURA, Y., CHIYODA, S., & TAKAKU, F. (1971) Myeloid Stem Cells in Various Hemopoietic Disorders. *International Symposium on Erythropoiesis*, Tokyo, 1974, p. 29.

MORRIS, T. C., McNEILL, T. A. & BRIDGES, J. M. (1975) Inhibition of Normal Human *in vitro* Colony Forming Cells by Cells from Leukaemic Patients. *Br. J. Cancer*, **31**, 641.

PIKE, B. L. & ROBINSON, W. A. (1970) Human Bone Marrow Colony Growth in Agar-gel. *J. cell. Physiol.*, **76**, 77.

SENN, J. S., McCULLOCH, E. A. & TILL, J. E. (1967) Comparison of Colony Forming Ability of Normal and Leukaemic Human Marrow in Cell Culture. *Lancet*, ii, 597.